# In vivo characterization of distinct modality-specific subsets of somatosensory neurons using GCaMP

**DOI:** 10.1126/sciadv.1600990

**Published:** 2016-11-11

**Authors:** Edward C. Emery, Ana P. Luiz, Shafaq Sikandar, Rán Magnúsdóttir, Xinzhong Dong, John N. Wood

**Affiliations:** 1 Molecular Nociception Group, Wolfson Institute for Biomedical Research, University College London, Gower Street, London WC1E 6BT, UK.; 2 Howard Hughes Medical Institute, Johns Hopkins University School of Medicine, Baltimore, MD 21205, USA.

**Keywords:** Pain modality, nociception, polymodal nociceptor, GCaMP imaging

## Abstract

Mechanistic insights into pain pathways are essential for a rational approach to treating this vast and increasing clinical problem. Sensory neurons that respond to tissue damage (nociceptors) may evoke pain sensations and are typically classified on the basis of action potential velocity. Electrophysiological studies have suggested that most of the C-fiber nociceptors are polymodal, responding to a variety of insults. In contrast, gene deletion studies in the sensory neurons of transgenic mice have frequently resulted in modality-specific deficits. We have used an in vivo imaging approach using the genetically encoded fluorescent calcium indicator GCaMP to study the activity of dorsal root ganglion sensory neurons in live animals challenged with painful stimuli. Using this approach, we can visualize spatially distinct neuronal responses and find that >85% of responsive dorsal root ganglion neurons are modality-specific, responding to either noxious mechanical, cold, or heat stimuli. These observations are mirrored in behavioral studies of transgenic mice. For example, deleting sodium channel Nav1.8 silences mechanical- but not heat-sensing sensory neurons, consistent with behavioral deficits. In contrast, primary cultures of axotomized sensory neurons show high levels of polymodality. After intraplantar treatment with prostaglandin E_2_, neurons in vivo respond more intensely to noxious thermal and mechanical stimuli, and additional neurons (silent nociceptors) are unmasked. Together, these studies define polymodality as an infrequent feature of nociceptive neurons in normal animals.

## Teaser

In vivo imaging shows that the great majority of somatosensory neurons are modality-specific.

## INTRODUCTION

Pain afflicts more than 1.5 billion people worldwide, with hundreds of millions suffering from long-term poorly treated pain (*1*). Peripheral drive from sensory neurons activated by tissue-damaging stimuli can cause pain, and local nerve block is very effective in most pain syndromes, focusing attention on somatosensory neurons as a good target for therapeutic intervention (*2*). A variety of noxious transduction receptors and channels have been identified by molecular cloning of sensory neuron transcripts, and attempts have been made to link sensation with specific receptor activation, which are not always successful (*3*). In 1941, Gasser (*4*) classified peripheral sensory neurons into three types on the basis of action potential velocity. Although all three types (A-β, A-δ, and C-fibers) may respond to noxious stimuli (*4*, *5*), the focus of most groups has been the set of C-fiber–associated neurons that seem to respond to multiple types of damaging stimuli, the so-called polymodal nociceptors. As well as polymodal nociceptors, electrophysiological recordings in human skin also report classes of C-fiber nociceptors that only respond to mechanical or heat stimuli (*6*). In 1996, Perl (*7*) demonstrated that injury or injection of inflammatory mediators can unmask a set of previously silent nociceptors. Studies of nociceptive neurons in vivo have been largely restricted to electrophysiological recordings from teased fibers, extracellular recordings, or recordings from the somata of individual dorsal root ganglion (DRG) neurons (*8*–*10*), whereas in vitro recordings also demonstrated polymodality (*11*, *12*). In contrast, numerous studies of transgenic mice in which particular cell types or molecular markers have been deleted are consistent with the existence of modality-specific sets of sensory neurons (*13*–*19*). How to reconcile these findings with the hypothesis that most of the nociceptors are polymodal is a major challenge. Here, using an in vivo GCaMP optical imaging approach to monitor neuronal responses to noxious stimuli in situ, we show that most of the somatosensory neurons in normal animals are modality-specific.

## RESULTS

### Assessment of GCaMP sensitivity in DRG neurons in vitro and in vivo

**Fig. 1:**
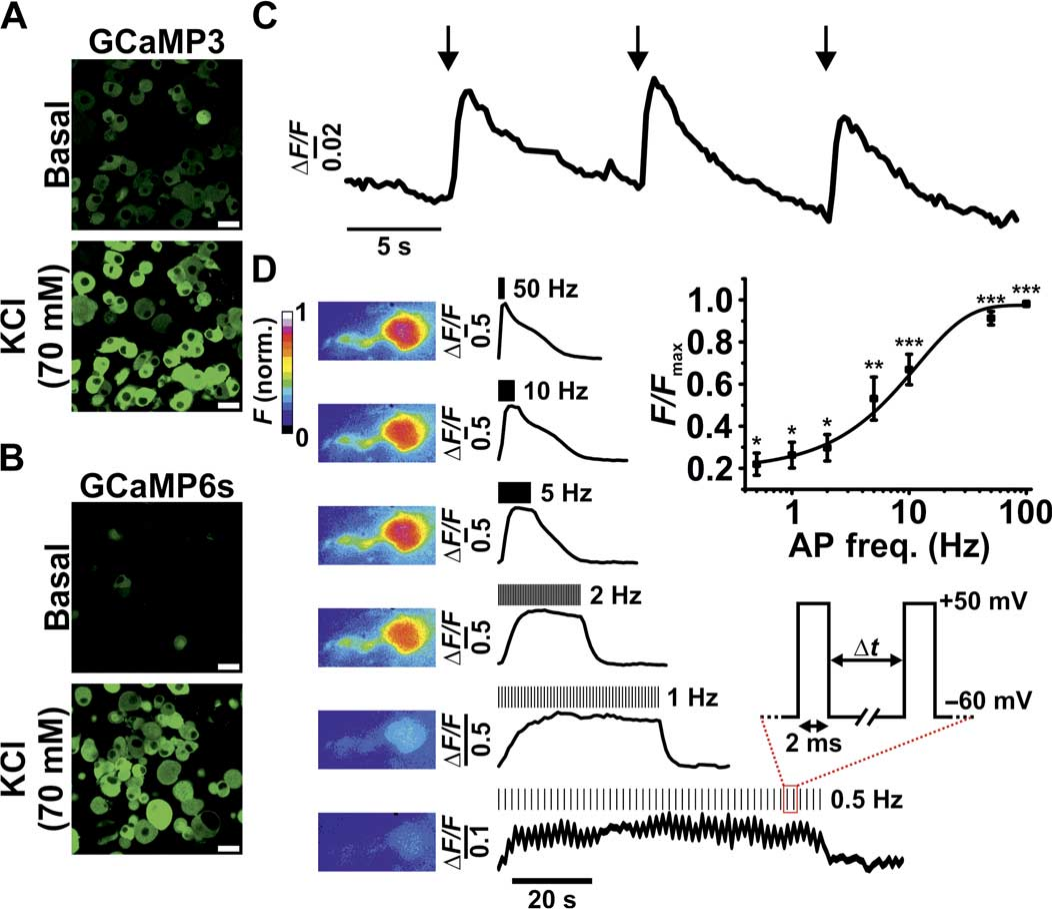
Assessment of GCaMP3 and GCaMP6s activity and sensitivity in DRG neurons in vitro. (**A** and **B**) Confocal images showing changes in fluorescence from GCaMP3-expressing (A) and GCaMP6s-expressing (B) DRG neurons following the application of KCl. Scale bars, 20 μm. (**C**) Fluorescence response profile of an individual GCaMP6s-expressing neuron in response to single depolarizing pulses. (**D**) Heatmap and corresponding fluorescence response of a single GCaMP3-expressing neuron in response to depolarizing pulses at variable frequency stimulations. Inset: Averaged responses shown as a function of frequency stimulation [action potential frequency (AP freq.) in hertz; *P < 0.05, **P < 0.01, and ***P < 0.001, repeated-measures analysis of variance (ANOVA); *n* = 7]. norm., normalized.

To determine the expression and sensitivity of the GCaMP indicators used, we cultured and studied DRG neurons expressing either GCaMP3 or GCaMP6s in vitro. The application of high extracellular KCl (70 mM) caused a detectable change in GCaMP fluorescence in 95% (1102 of 1158) and 97% (1311 of 1352) of GCaMP3- and GCaMP6s-expressing DRG neurons, respectively (Fig. 1, A and B). Using electrophysiological stimulation in vitro, we found that applying a single action potential–like stimulus caused a detectable change in GCaMP fluorescence (Fig. 1C and movie S1) and that a train of stimuli (50 pulses) was detectable at frequencies ≥0.5 Hz (Fig. 1D and movies S2 to S4).

**Fig. 2:**
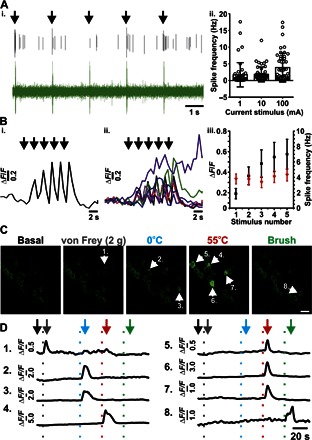
Assessment of GCaMP3 activity and sensitivity in DRG neurons in vivo. (**Ai**) Extracellular electrophysiological recording from an intact L4 DRG in vivo showing individual action potentials being elicited by brief (20 ms) 100-mA pulses (denoted by black arrows). The upper black trace shows activity from a single neuron extracted from the raw signal (green). (**Aii**) Summary of the average number of action potentials elicited from individual neurons in response to 1-, 10-, and 100-mA pulses (20 ms; *n* = 48). (**Bi**) Example fluorescence trace of a single GCaMP3 neuron in response to a train of 100-mA pulses (20 ms, 0.5 Hz). Responses from all neurons analyzed are shown in (**Bii**) (*n* = 7). (**Biii**) Summary of the average change in fluorescence (Δ*F*/*F*) for each GCaMP3 neuron for sequential current stimulations (100 mA, 20 ms, 0.5 Hz). The average number of action potentials elicited per current injection is overlaid in red (*n* = 48). (**C**) In vivo confocal images showing the levels of GCaMP3 fluorescence from L4 DRG neurons in response to the stimuli applied to the plantar surface. Numbered arrows indicate activated neurons within each application frame. Scale bar, 20 μm. (**D**) Raw traces of each neuron labeled with a numbered arrowhead in (C). The vertical dotted lines represent the points at which the stimuli were applied to the plantar surface. Gray, von Frey; blue, 0°C water; red, 55°C water; green, light brush. The arrows mark the points at which the frames in (C) were taken for each stimulus event.

Next, we wanted to investigate the sensitivity of GCaMP3 in vivo. Anesthetized mice expressing GCaMP3 had their L4 DRG exposed and were imaged using laser scanning confocal microscopy. Transient stimulating current pulses (5 × 20 ms at 0.5 Hz) of varying amplitude (1, 10, and 100 mA) were applied to the plantar surface of the ipsilateral hind paw, and the activity at the level of the DRG was recorded using in vivo electrophysiology (Fig. 2Ai). We found that transient current pulses of 1, 10, and 100 mA led to the generation of 1.72 (±0.28), 1.83 (±0.18), and 3.88 (±0.33) action potentials at the level of the DRG, respectively (Fig. 2Aii). The proportions of nonresponding DRGs to individual current pulses were 68.75% (165 of 240), 47.92% (115 of 240), and 38.33% (92 of 240) for 1, 10, and 100 mA stimuli, respectively. Because 100 mA was the most reliable in stimulating DRG neurons, this stimulus was used to study the changes in GCaMP3 activity in vivo. Current stimulation caused a detectable change in GCaMP3 fluorescence from DRG neurons (Fig. 2B, i and ii), with repetitive stimulation causing a gradual rise in overall fluorescence, most likely reflecting the slow off-kinetics of the GCaMP3 rather than a change in action potential frequency (Fig. 2Biii). Next, we tested the sensitivity of GCaMP3 to a variety of noxious and tactile stimuli applied to the plantar surface of the ipsilateral hind paw. Stimuli, including brush, noxious mechanical, hot (55°C), and cold (0°C) stimuli caused the discrete activation of L4 DRG neurons and could all be readily detected (Fig. 2, C and D).

### Basal sensory modality testing in mice expressing GCaMP3 and GCaMP6s

**Fig. 3:**
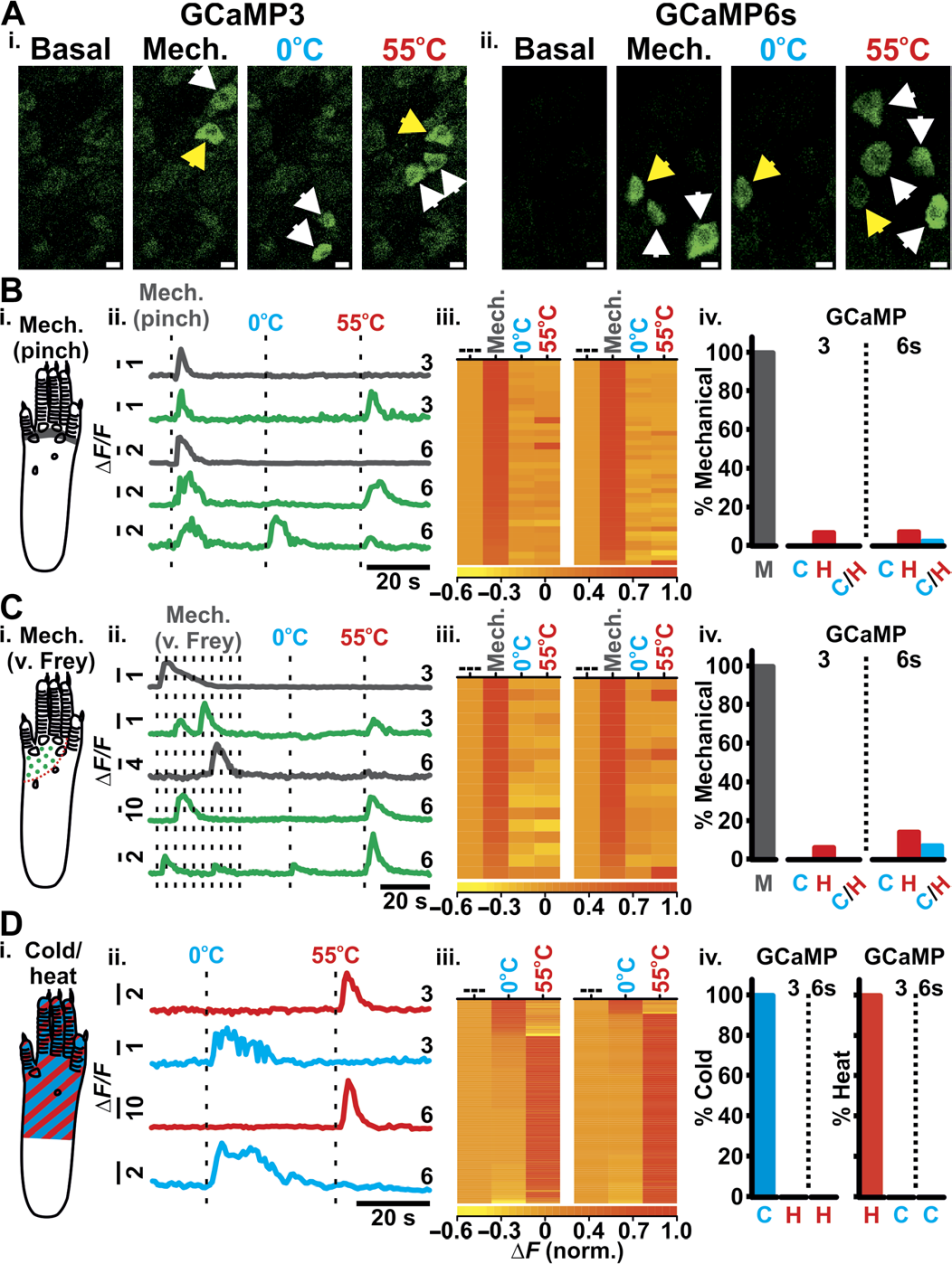
Basal sensory modality testing in mice expressing GCaMP3 and GCaMP6s. (**A**) In vivo confocal images showing levels of GCaMP3 (**Ai**) and GCaMP6s (**Aii**) fluorescence from L4 DRG neurons at rest (basal) and in response to mechanical (pinch), cold (0°C), and heat (55°C) stimuli applied to the plantar surface. Unimodal (white arrowheads) and polymodal (yellow arrowheads) neurons are shown. Scale bars, 10 μm. Diagram of application area of the pinch stimulus (**Bi**) (gray), von Frey stimuli (**Ci**) (green dots), and cold/heat stimuli (**Di**) (red/blue stripes). Example raw traces from L4 DRG neurons in response to mechanical [(**Bii**) pinch; (**Cii**) von Frey] and 0° and 55°C stimuli (dotted lines indicate application of stimuli; 3 and 6 refer to GCaMP3 and GCaMP6s traces, respectively). Gray- and green-colored traces indicate modality-specific and polymodal response profiles, respectively. (**Dii**) Example raw traces from L4 DRG neurons in response to thermal stimuli (dotted lines indicate application of stimuli) (3, GCaMP3; 6, GCaMP6s). Red- and blue-colored traces indicate neurons responding to 55° and 0°C stimuli, respectively. Heatmap response profiles for every DRG neuron tested for mechanical [(**Biii**) pinch; (**Ciii**) von Frey] and 0° and 55°C stimuli for both GCaMP3-expressing (left; *n* = 17 to 31) and GCaMP6s-expressing neurons (right; *n* = 17 to 45). (**Diii**) Heatmap response profiles for every neuron responsive to 0° or 55°C stimuli for both GCaMP3-expressing (left; *n* = 146) and GCaMP6s-expressing (right; *n* = 100) neurons. Summary of DRG neurons [M, mechanical; (**Biv**) pinch; (**Civ**) von Frey] also responding to 0°C (C), 55°C (H), or both 0° and 55°C (C/H) stimuli from mice expressing GCaMP3 or GCaMP6s, respectively. (**Div**) Summary of cold-sensitive neurons also responding to a heat stimulus and of heat-sensitive neurons also responding to a cold stimulus from mice expressing GCaMP3 or GCaMP6s, respectively. Recordings were made from 13 animals expressing GCaMP3 and 8 animals expressing GCaMP6s of both sex.

**Fig. 4:**
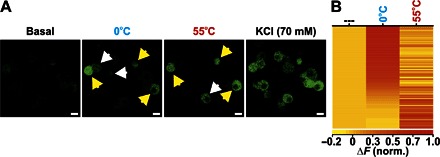
Assessment of cold and heat sensitivity in cultured DRG neurons in vitro. (**A**) In vitro images showing changes in GCaMP3 fluorescence from cultured DRG neurons at rest (basal) and in response to perfused 0°C extracellular solution, 55°C extracellular solution, and KCl. Unimodal (white arrowheads) and polymodal (yellow arrowheads) neurons are shown. Scale bars, 10 μm. (**B**) Heatmap response profiles for every neuron responsive to 0° or 55°C stimuli. Each row displays the maximum normalized fluorescent signal acquired during each application window (*n* = 171).

Next, we investigated the basal modality responses of L4 DRG neurons from GCaMP3- and GCaMP6s-expressing mice. Both GCaMP3 and GCaMP6s indicators were capable of detecting different noxious stimuli, including mechanical force [either applied with serrated forceps (pinch) or 2-g von Frey filament], cold (0°C water), or heat stimuli (55°C water) (Fig. 3A, i and ii, and movie S5). Each stimulus was applied to the ipsilateral hind paw, as shown in Fig. 3 (Bi, Ci, and Di). The application area for cold and heat stimuli was made much larger than the mechanical stimulus area to ensure that all mechanically sensitive neurons could be investigated for their responses to 0° and 55°C stimuli. In response to mechanical pinch stimulation, only 6.45% (2 of 31) of GCaMP3-expressing and 6.67% (3 of 45) of GCaMP6s-expressing DRG neurons responded to both mechanical and heat stimulation, and only 2.22% (1 of 45) of GCaMP6s neurons responded to mechanical, cold, and heat stimulation (Fig. 3B, iii and iv). A similar result was observed when von Frey stimulation was used in place of mechanical pinch, with 5.88% (1 of 17) of GCaMP3 and 11.76% (2 of 17) of GCaMP6s neurons responding to both mechanical and heat stimulation, and only 5.88% (1 of 17) of GCaMP6s neurons responding to mechanical, cold, and heat stimulation. Of the GCaMP3 and GCaMP6s neurons that only respond to thermal stimuli (potentially having receptive fields outside of the mechanical stimulation area), all were modality-specific, responding to either cold or hot stimulation (*n* = 146 or 100, respectively; Fig. 3D). These observations contrast with the findings from similar experiments conducted on cultured DRG neurons in vitro, where 19.88% (34 of 171) of neurons are cold-responsive, 26.32% (45 of 171) of neurons are heat-responsive, and 53.80% (92 of 171) are both cold- and heat-responsive (Fig. 4, A and B).

**Fig. 5:**
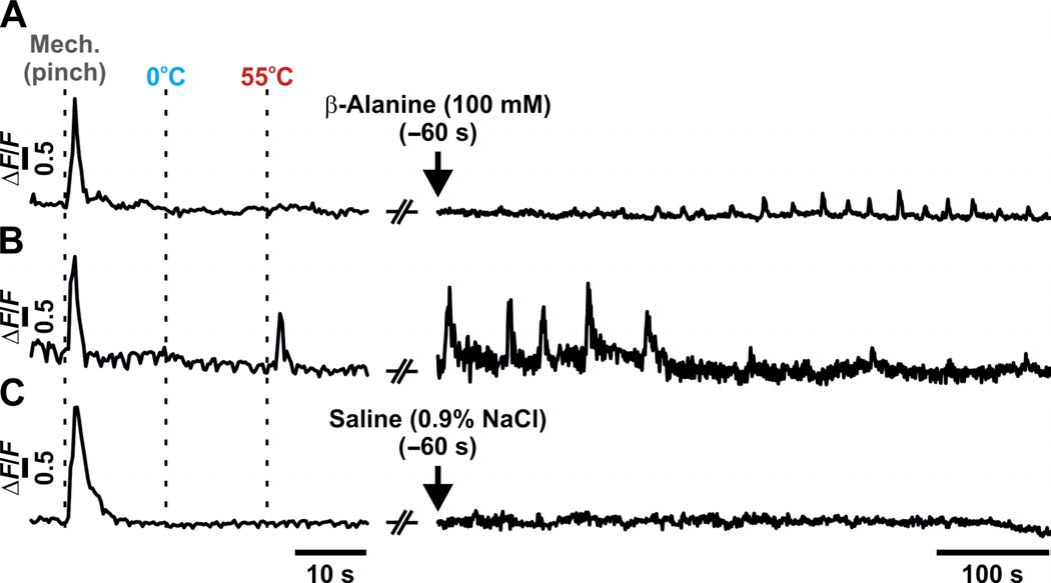
Assessment of sensory modality responses of β-alanine–sensitive neurons in vivo. Example GCaMP3 traces of individual DRG neurons following the application of modality-specific stimuli (mechanical pinch, 0°C water, or 55°C water) and their respective sensitivity to the intraplantar administration of β-alanine (100 mM). Most of the β-alanine–sensitive neurons (*n* = 5 or 7) were only responsive to mechanical stimuli (**A**), with few responding to both mechanical and heat stimuli (**B**) (*n* = 2 or 7). A typical trace following saline (0.9% NaCl) administration is also shown (**C**).

It has been reported that β-alanine–sensitive mas-related G protein–coupled receptor member D–positive (MrgprD^+^) neurons are responsive to both noxious heat and mechanical stimuli (*20*); however, behaviorally, MrgprD^+^ neurons only appear to be required for noxious mechanical sensitivity rather than thermal stimuli (*19*). To further consolidate our findings, we injected β-alanine (100 mM), a specific activator of MrgprD^+^ neurons, into the ipsilateral hind paw and recorded changes in GCaMP fluorescence from DRG neurons. Of the seven mechanically sensitive neurons that responded to β-alanine, five of them were unresponsive to noxious heat, and only two responded to both mechanical and heat stimulation, consistent with the principal role of MrgprD^+^ neurons in noxious mechanosensation (Fig. 5, A and B) (*19*). No spontaneous activity was observed following the injection of saline (Fig. 5C).

### Effect of inflammatory agents on the sensory modalities of DRG neurons in vivo

**Fig. 6:**
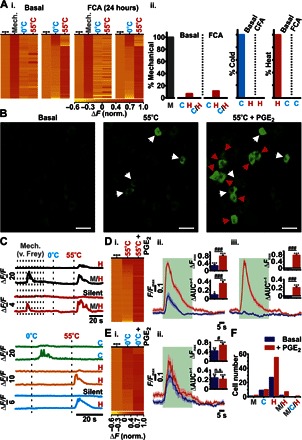
The effect of inflammatory agents on the sensory modalities response of DRG neurons in vivo. (**Ai**) Heatmap response profiles for individual GCaMP3-expressing mechanically sensitive (pinch) neurons tested for sensitivity toward mechanical (pinch), 0°, and 55°C stimuli (left), as well as neurons responsive to 0° or 55°C stimuli (right), before (basal; *n* = 31 and *n* = 146, respectively) or 24 hours after administration of FCA (*n* = 90 or *n* = 102, respectively). (**Aii**) Summary of mechanically sensitive neurons responding to thermal stimuli as well as thermal-sensitive neurons responding to 55° or 0°C before and 24 hours after the administration of FCA, respectively. Baseline (*n* = 13 animals) and after FCA (*n* = 10 animals). (**B**) In vivo confocal images showing levels of GCaMP3 fluorescence from L4 DRG neurons that respond to 55°C before (pre-PGE_2_) and 10 min after administration of PGE_2_ (55°C + PGE_2_). Basally responsive (white arrowheads) and basally silent (red arrowheads) neurons are shown. Scale bars, 50 μm. (**C**) Example raw traces of neuronal modality profiles before and after PGE_2_ administration. (**Di**) Heatmap response profile for individual neurons responsive to 55°C before or after PGE_2_ administration. Trace showing the average change in GCaMP3 fluorescence following peripheral noxious heat stimulation (green application bar) from basally heat-responsive neurons (**Dii**) or basally silent neurons (**Diii**). AUC, area under the curve. (**Ei**) Heatmap response profile for individual neurons responsive to 0°C before or after PGE_2_ administration. (**Eii**) Trace showing the average change in GCaMP3 fluorescence following peripheral noxious cold stimulation (0°C; green application bar) from basally cold-responsive neurons. Changes in relative maximal fluorescence and AUC (insets). n.s., not significant. (**F**) Summary of the number of cells displaying discrete modalities before and after PGE_2_ administration (*n* = 75) [*P < 0.05, **P < 0.01, and ***P < 0.001 (comparisons made to baseline); #*P* < 0.05 and ###*P* < 0.001 (comparisons made between groups)]. Pre- and post-PGE_2_ recordings were made from five animals. M, mechano; C, cold; H, heat; M/H, mechano-heat; M/C/H, mechano-cold-heat; C/H, cold and hot.

Next, we investigated whether the sensory modalities of DRG neurons are altered during inflammation. To test this, we injected Freund’s complete adjuvant (FCA) into the plantar surface of the ipsilateral hind paw and recorded stimulus-evoked GCaMP3 activity from L4 DRG neurons in vivo after 24 hours. We observed a small but statistically insignificant increase in the relative number of polymodal neurons responsive to mechanical (pinch) and heat (55°C) stimuli following FCA administration (10.23%, 9 of 88) (Fig. 6A, i and ii, and movie S6). Neurons responding only to thermal stimuli (cold or heat) were modality-specific (Fig. 6A). To extend this analysis so that we could examine the responsive cells and their sensory modalities in a single neuronal field over a shorter time course, we gave an intraplantar injection of prostaglandin E_2_ (PGE_2_) and recorded from the same neurons before and 10 min after its administration (Fig. 6B). Following PGE_2_ administration, we observed a change in the modality of numerous L4 DRG neurons (Fig. 6C), as well as augmented responses from heat- and cold-sensitive neurons (Fig. 6, D and E, and movies S7 and S8). Furthermore, we also observed responses from neurons that were previously unresponsive (36 of 75 neurons). Although the number of cold-sensitive neurons only increased modestly (basal, *n* = 9; PGE_2_, *n* = 10), we observed a much bigger increase in the number of neurons responsive to mechanical (von Frey; basal, *n* = 1; PGE_2_, *n* = 2), heat (55°C; basal, *n* = 27; PGE_2_, *n* = 55), and mechanical and heat stimulation (polymodal; basal, *n* = 0; PGE_2_, *n* = 1) (Fig. 6E).

### In vivo assessment of modality responses following Na_V_1.8 deletion

Finally, we compared the properties of wild-type sensory neurons with those that no longer express the voltage-gated sodium channel Na_V_1.8 (Na_V_1.8^−/−^). In vivo GCaMP3 recordings of Na_V_1.8^−/−^ DRG showed that only 5.19% (4 of 77) of neurons that gave a response to any stimulus were mechanically sensitive, in comparison to wild-type neurons, where 32.95% (29 of 88) of neurons were mechanically sensitive (see fig. S1). In contrast, cold, heat, and mechano-heat–sensitive neurons were similar between wild-type and Na_V_1.8^−/−^ mice (see fig. S1). Na_V_1.8-null mutant mice are mechano-insensitive but have normal heat pain thresholds (*21*).

## DISCUSSION

Technical advances have often presaged new biological insights (*22*). The development of GCaMP indicators as markers for neuronal activity has provided the first practicable route to analyze neuronal activity in vivo without interfering with the intracellular milieu or without penetrating the cell with an electrode (*23*, *24*). Here, we show that low-frequency action potential firing can give rise to detectable changes in GCaMP fluorescence, enabling us to measure spatially distinct response profiles of large populations of sensory neurons to different tactile and noxious stimuli in vivo. These data demonstrate that neurons responsive to both mechanical and heat stimuli are restricted to ~10% of the neurons analyzed, whereas a response to mechanical, heat, and cold stimuli occurs only in ~5% of neurons.

What are the consequences of these observations? Primarily, they suggest that the basal phenotype of a sensory neuron is potentially affected by the recording technique used. Most of the studies investigating sensory neuron function to date have used electrophysiological approaches, either in ex vivo, in vitro, or in vivo models, at the level of the afferent nerve or the soma. Although electrophysiological approaches provide unrivaled sensitivity, they are not without compromise.

Ex vivo models that rely on the transection and isolation of primary afferent nerves will inevitably perturb the physiological integrity of the system being investigated, potentially affecting how it responds to noxious stimuli. A similar argument can also be made for the recordings made from sensory neurons in vitro. Cultured sensory neurons represent a population of axotomized and dissociated cells that have been maintained under quasi-physiological conditions that typically favor the viability of small neurons through the use of specific growth factors, such as nerve growth factor. Furthermore, responses of neurons in vitro are likely to be a fairer representation of soma sensitivity rather than those of afferent terminals, which could explain why >50% of cultured neurons showed polymodality toward both heat and cold stimuli, a phenotype we did not observe in vivo. It is possible that cell somata are intrinsically polymodal, reflecting protein synthesis of transducing receptors for noxious stimuli in the cell body, whereas peripheral terminals in vivo are modality-specific.

Although potential artifacts caused by nerve/neuron isolation, either in ex vivo or in vitro models, may offer an explanation for the differing observations between optical imaging and electrophysiological recording, this still does not reconcile the imaging data with the data obtained from in vivo electrophysiological studies where the integrity of peripheral pain pathways is not compromised (*25*). However, extracellular recording potentially provides data from more than one sensory neuron, and the same problem may occur with teased fiber recordings, so this may contribute to the higher frequency of polymodality observed with these techniques.

One intriguing observation made from the current study is that the modality of a neuron in vivo can be altered rapidly by inflammatory mediators, such as PGE_2_. Sensory neuron subsets express different levels of E prostanoid (EP) receptors and protein kinase A isoforms, which may result in different responses to PGE_2_ in different sets of sensory neurons (*26*). In addition to an increase in the intensity of neuronal responses following PGE_2_ to heat and cold stimuli, we also observed that PGE_2_ administration unmasked a significant population of previously unresponsive neurons or silent nociceptors, a population that was almost equal in size to the basally responsive population. This phenomenon occurred within minutes of administration, supporting posttranslational regulation at the peripheral terminals of sensory neurons. There is strong evidence for second-messenger regulation of excitability in sensory neurons, with effects of diacylglycerol and cyclic adenosine monophosphate on kinases and guanosine 5′-triphosphate exchange factors leading to biophysical changes in the properties of ion channels, such as *I*_h_, and enhanced trafficking of ion channels and receptors, all of which may contribute to enhanced neuronal excitability (*26*, *27*). This observation could reconcile any discrepancy in the number of polymodal neurons reported. It could be argued that the more invasive an approach is, the more likely it is to cause the release of inflammatory mediators, such as PGE_2_, which could act to change the basal modality responses of sensory neurons, as we have observed in the current study.

Although the release of inflammatory mediators may offer an explanation for the differences in the observed polymodality, it is nevertheless important to consider the potential limitations associated with GCaMP imaging. First, the temporal resolution of this approach is much lower than that obtained using an electrophysiology-based alternative, and although we can reliably detect low-level action potential firing in vitro and in vivo, little information can be gained about action potential number, frequency, or conduction velocity. Second, the spatial resolution, although still superior to electrophysiology-based techniques, is limited to a single plane in the current study, potentially limiting the population size (and type) of the neurons studied. An additional point to consider is that GCaMP expression, and therefore sensitivity, at the level of the somata may be variable between neurons. Numerous experiments were performed to test the sensitivity of the GCaMP probe in vitro and in vivo; however, potential variation in GCaMP expression could affect how the activation of a neuronal afferent is represented by fluorescence at the level of the DRG.

In summary, our in vivo observations of intact peripheral sensory neuron function using noninvasive optical imaging techniques contrast with the data obtained by electrophysiological techniques. It seems clear that the prevailing view of a significant role for C-fiber–associated polymodal nociceptors in normal animals needs revision. The exquisite sensitivity of GCaMP to detect low-level action potential firing demonstrates the modality-specific function of most somatosensory neurons in uninjured animals. This finding is consistent with the many examples of modality-specific deficits described in transgenic knockout mouse studies of pain behavior (*28*). This technology should allow us to identify sets of sensory neurons that are activated in different pain states and, using RNA sequencing analysis, define subsets of neurons that may be associated with distinct pain conditions (*29*). The ability to distinguish pain mechanisms that may provide similar phenotypes through distinct sets of neurons is, in the long term, an important route to rational human pain treatment when markers for these mechanisms have been identified.

## MATERIALS AND METHODS

### Animals

All animal procedures were approved by local ethical review committees and conformed to U.K. Home Office regulations. These guidelines meet international standards defined by the European Community. Experiments were performed using heterozygous GCaMP3- and GCaMP6s-expressing mice (8 to 10 weeks old; male and female) on a C57BL/6 background. *Pirt*-GCaMP3 mice were generated by X.D. (Johns Hopkins University, Baltimore, MD) (*30*). Rosa26-floxed-STOP-GCaMP6s mice were crossed with Advillin-Cre mice to enable DRG-specific expression of GCaMP6s (The Jackson Laboratory). Mice deficient in Na_V_1.8 expression were previously described (*31*).

### Cell culture

DRG neurons were isolated from GCaMP3- and GCaMP6s-expressing mice (see “Animals”). Briefly, the dorsal side of the spinal column was cut away using dissecting scissors, and the spinal cord was removed. DRGs were dissected from the entire length of the spinal column and transferred into a preequilibrated solution of Hanks’ balanced salt solution containing collagenase (type XI; 5 mg/ml), dispase (10 mg/ml), Hepes (5 mM), and glucose (10 mM) for 30 min (37°C, 5% CO_2_). DRGs were then gently centrifuged at 100 relative centrifugal force (rcf) for 2 min, and the supernatant was discarded and replaced with warmed Dulbecco’s modified Eagle’s medium (DMEM), supplemented with l-glutamine (1%), glucose (4.5 g/liter), and sodium pyruvate (110 mg/liter) supplemented with 10% fetal bovine serum (FBS). The DRGs were mechanically triturated using fire-polished glass Pasteur pipettes. The dissociated cells were then centrifuged at 100 rcf for 8 min, and the supernatant was removed and replaced with an appropriate volume of DMEM supplemented with FBS and nerve growth factors (50 ng/ml). Cells were plated onto sterilized glass coverslips (13 mm, size 0) precoated with poly-l-lysine (1 mg/ml) and laminin (1 mg/ml). Cells were maintained at 37°C for 15 to 40 hours before use at 5% CO_2_.

### In vitro electrophysiology

All electrophysiological experiments were performed using an AxoPatch 200B Amplifier and the DigiData 1322A Digitizer (Axon Instruments), controlled by Clampex software (version 10, Molecular Devices). Filamented borosilicate microelectrodes (GC150TF-15, Harvard Apparatus) were fire-polished to give a final resistance of 2.5 to 4 megohms. For voltage-clamp experiments, the following solutions were used: the extracellular solution contained 140 mM NaCl, 3 mM KCl, 1 mM MgCl_2_, 2 mM CaCl_2_, 10 mM Hepes, 1 mM glucose (pH 7.4) with NaOH; the intracellular solution contained 140 mM KCl, 5 mM NaCl, 1.6 mM MgCl_2_, 10 mM HEPES (pH 7.3) with KOH. To avoid dialysis of GCaMP indicators, amphotericin B (200 μg/ml) was added to the intracellular solution on the day of experimentation to enable perforated patch configuration. After achieving perforated patch configuration, a holding potential of −60 mV was applied, and the series resistance was compensated by ≥70%.

### In vivo electrophysiology

Mice were anesthetized using ketamine (120 mg/kg) (Fort Dodge Animal Health Ltd.) and medetomidine (1.2 mg/kg) (Orion Pharma) and secured in a stereotaxic frame. A lateral laminectomy was performed to expose lumbar DRG, and the extracellular recordings were made for L4 DRG neurons (14- to 53-μm depth) using a Parylene-coated tungsten electrode (A-M Systems) in C57BL/6 mice (*n* = 48 neurons). Transcutaneous electrical stimulation was delivered with bipolar electrodes at 20-ms-wide pulses at increasing intensity (1, 10, and 100 mA), and the evoked activity was visualized on an oscilloscope and discriminated on a spike amplitude and waveform basis using a CED 1401 interface coupled to Spike2 software (Cambridge Electronic Design).

### In vitro GCaMP imaging

GCaMP3- and GCaMP6s-expressing DRG neurons were cultured as described (see “Cell culture”), placed into a perfusion chamber attached to an inverted microscope (Nikon TE300) for electrophysiology experiments or an upright confocal microscope (Leica SP8) for thermal sensitivity experiments, and imaged using an oil immersion objective (40×) or dipping objective (25×), respectively. During each imaging experiment, the neurons were continuously perfused with extracellular solution (see “In vitro electrophysiology”) using a gravity-fed perfusion system. For rapid heating/cooling of the extracellular perfusate, a Peltier-driven in-line perfusion outlet was used (Warner Instruments). The fluorescence dynamics of GCaMP3/6s (excitation, 480 ± 10 nm) were measured within each cell using a monochromator (Cairn Research) and an EXi AQUA digital camera (QImaging). Images were acquired at 0.1 to 1 Hz and were analyzed, after background subtraction, using MetaFluor software (Molecular Devices).

### In vivo GCaMP imaging

Heterozygous GCaMP3- and GCaMP6s-expressing mice (8 to 10 weeks old; male and female) were anesthetized using ketamine (120 mg/kg) (Fort Dodge Animal Health Ltd.) and medetomidine (1.2 mg/kg) (Orion Pharma). The depth of anesthesia was assessed by pedal reflexes, breathing rate, and whisker movement. Throughout the experiment, the body temperature of the animal was maintained at 37°C using a heated mat (VetTech). A dorsal laminectomy was performed at spinal level L3-L5. Briefly, the skin was incised longitudinally, and the paravertebral muscles were cut to expose the vertebral column. Transverse and superior articular processes of the vertebra were removed using microdissection scissors and OmniDrill 35 (WPI). To have a clean image of the sensory cell bodies neurons, the dura mater and the arachnoid membranes were carefully opened using microdissection forceps. The animal was mounted onto a custom-made clamp that attached to the vertebral column (L1), rostral to the laminectomy. The trunk of the animal was slightly elevated to reduce interference caused by respiration. The artificial spinal fluid [containing 120 mM NaCl, 3 mM KCl, 1.1 mM CaCl_2_, 10 mM glucose, 0.6 mM NaH_2_PO_4_, 0.8 mM MgSO_4_, 18 mM NaHCO_3_ (pH 7.4) with NaOH] was constantly perfused over the exposed DRG during the procedure to maintain tissue integrity. All in vivo imaging experiments were performed using a Leica SP8 confocal microscope (Leica). For GCaMP excitation, a laser wavelength at 488 nm (2% laser power) was used, and the images were acquired at a bidirectional scan speed of 800 Hz. Noxious and tactile stimulation was applied to the left hind paw (ipsilateral to the exposed DRG). Noxious mechanical stimulation was performed using either serrated forceps (pinch) or von Frey filament (2 g) stimulation. The site of stimulation is illustrated in Fig. 2. Tactile mechanical stimulation was performed using a fine brush applied to the plantar surface of the hind paw (same area as for the thermal stimuli). Thermal stimulation was performed by transiently immersing the hind paw with cooled (0°C) or heated (55°C) water. The application of 37°C water did not evoke any change in neuronal activity.

### Inflammatory models

To induce a model of long-lasting inflammation, 20 μl of FCA was applied in the ipsilateral hind paw of the mouse. Edema was confirmed 24 hours later, followed by the dissection and imaging of the animal. Acute inflammation was induced with PGE_2_ (10 nmol per paw) injected into the ipsilateral hind paw of the mouse.

### Image analysis

All in vivo data were analyzed using LASX analysis software (Leica). For normalized responses, each raw trace had the minimum trace value subtracted and was then normalized to the maximum trace value [(*F* − *F*_min_)/(*F*_max_ − *F*_min_)] to get a signal range of 0 to 1. Heatmap summaries were generated by subtracting the maximum normalized baseline value from each trace to give a baseline value of 0. To determine whether a neuron was responsive to a given stimulus, the raw traces were first smoothed by averaging the preceding five frames of any test frame to reduce noise. Then the derivative of each frame was taken as Δ*F*/Δ*t*. The neuron was included as a responder to a given stimulus if Δ*F*_stim_/Δ*t* > [(Δ*F*_basal_/Δ*t*) + 4σ_basal_], where *F*_stim_ is the maximum derivative value within a given application window, *F*_basal_ is the average derivative baseline value, and σ_basal_ is the SD of the baseline derivative values. Where appropriate, statistical analysis was performed using repeated-measures ANOVA with Bonferroni post hoc testing, unless otherwise stated. Statistical analysis was performed in GraphPad Prism 6.0.

## Supplementary Material

http://advances.sciencemag.org/cgi/content/full/2/11/e1600990/DC1

## SUPPLEMENTARY MATERIALS

Supplementary material for this article is available at http://advances.sciencemag.org/cgi/content/full/2/11/e1600990/DC1 fig. S1. In vivo assessment of modality responses following Na_V_1.8 deletion. movie S1. Neuronal activation assessed by GCaMP6s fluorescence in vitro. movie S2. Frequency-dependent (0.5 Hz) changes in neuronal activation assessed by GCaMP3 fluorescence. movie S3. Frequency-dependent (1 Hz) changes in neuronal activation assessed by GCaMP3 fluorescence. movie S4. Frequency-dependent (10 Hz) changes in neuronal activation assessed by GCaMP3 fluorescence. movie S5. Neuronal activation assessed by GCaMP3 fluorescence in vivo. movie S6. Neuronal activation following FCA treatment assessed by GCaMP3 fluorescence in vivo. movie S7. Neuronal activation before PGE_2_ treatment assessed by GCaMP3 fluorescence in vivo. movie S8. Neuronal activation following PGE_2_ treatment assessed by GCaMP3 fluorescence in vivo.
