# Supplementary material for: In vivo characterization of distinct modality-specific subsets of somatosensory neurons using GCaMP
Source: Sci Adv. 2016 Nov 11;2(11):e1600990. doi: 10.1126/sciadv.1600990 (PMC5106201; doi:10.1126/sciadv.1600990)
Supplement: http://advances.sciencemag.org/cgi/content/full/2/11/e1600990/DC1 [file supp_2_11_e1600990__index.html]

Science Advances | Science Advances

## Supplementary Materials

**This PDF file includes:**

- fig. S1. In vivo assessment of modality responses following NaV1.8 deletion.
- Legends for movies S1 to S8

Download PDF

**Other Supplementary Material for this manuscript includes the following:**

- movie S1 (.avi format). Neuronal activation assessed by GCaMP6s fluorescence in vitro.
- movie S2 (.avi format). Frequency-dependent (0.5 Hz) changes in neuronal activation assessed by GCaMP3 fluorescence.
- movie S3 (.avi format). Frequency-dependent (1 Hz) changes in neuronal activation assessed by GCaMP3 fluorescence.
- movie S4 (.avi format). Frequency-dependent (10 Hz) changes in neuronal activation assessed by GCaMP3 fluorescence.
- movie S5 (.avi format). Neuronal activation assessed by GCaMP3 fluorescence in vivo.
- movie S6 (.avi format). Neuronal activation following FCA treatment assessed by GCaMP3 fluorescence in vivo.
- movie S7 (.avi format). Neuronal activation before PGE2 treatment assessed by GCaMP3 fluorescence in vivo.
- movie S8 (.avi format). Neuronal activation following PGE2 treatment assessed by GCaMP3 fluorescence in vivo.

**Files in this Data Supplement:**

- Adobe PDF - 1600990\_SM.pdf
