# Supplementary material for: In vivo characterization of distinct modality-specific subsets of somatosensory neurons using GCaMP
Source: Sci Adv. 2016 Nov 11;2(11):e1600990. doi: 10.1126/sciadv.1600990 (PMC5106201; doi:10.1126/sciadv.1600990)
Supplement: http://advances.sciencemag.org/cgi/content/full/2/11/e1600990/DC1 [file 1600990_SM.pdf]

Science Advances

AAAS

advances.sciencemag.org/cgi/content/full/2/11/e1600990/DC1

Supplementary Materials for

# In vivo characterization of distinct modality-specific subsets of somatosensory neurons using GCaMP

Edward C. Emery, Ana P. Luiz, Shafaq Sikandar, Rán Magnúsdóttir, Xinzhong Dong, John N. Wood

Published 11 November 2016, *Sci. Adv.* **2**, e1600990 (2016)
DOI: 10.1126/sciadv.1600990

**The PDF file includes:**

- fig. S1. In vivo assessment of modality responses following $Na_V1.8$ deletion.
- Legends for movies S1 to S8

**Other Supplementary Material for this manuscript includes the following:**
(available at advances.sciencemag.org/cgi/content/full/2/11/e1600990/DC1)

- movie S1 (.avi format). Neuronal activation assessed by GCaMP6s fluorescence in vitro.
- movie S2 (.avi format). Frequency-dependent (0.5 Hz) changes in neuronal activation assessed by GCaMP3 fluorescence.
- movie S3 (.avi format). Frequency-dependent (1 Hz) changes in neuronal activation assessed by GCaMP3 fluorescence.
- movie S4 (.avi format). Frequency-dependent (10 Hz) changes in neuronal activation assessed by GCaMP3 fluorescence.
- movie S5 (.avi format). Neuronal activation assessed by GCaMP3 fluorescence in vivo.
- movie S6 (.avi format). Neuronal activation following FCA treatment assessed by GCaMP3 fluorescence in vivo.
- movie S7 (.avi format). Neuronal activation before $PGE_2$ treatment assessed by GCaMP3 fluorescence in vivo.
- movie S8 (.avi format). Neuronal activation following $PGE_2$ treatment assessed by GCaMP3 fluorescence in vivo.

**Supplementary Materials**

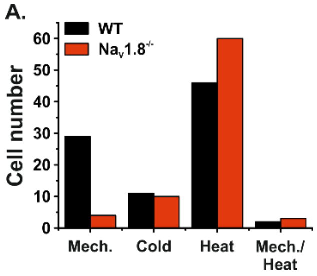


**fig. S1. In vivo assessment of modality responses following $Na_V1.8$ deletion. A.** Summary of the number of L4 DRG neurons recorded responding to different noxious stimuli including mechanical pinch (Mech.), 0°C water (Cold), 55°C water (Heat) as well as those responding to both mechanical and heat (Mech./Heat), from both WT and $Na_V1.8^{-/-}$ GCaMP3-expressing mice (neurons recorded: n = 88 for WT and n = 77 for $Na_V1.8^{-/-}$).

**movie S1. Neuronal activation assessed by GCaMP6s fluorescence in vitro.** Changes in GCaMP6s fluorescence from a DRG neuron in vitro in response to single depolarizing pulses (2 ms from −60 mV to +50 mV). The frame sequence (169 frames) has been accelerated for illustrative purposes (original acquisition rate $10.s^{-1}$).

**movies S2 to S4. Frequency-dependent changes in neuronal activation assessed by GCaMP3 fluorescence.** Changes in GCaMP3 fluorescence from cultured DRG neurons in response to a train (50 pulses) of action potential-like stimuli (2 ms from −60 mV to +50 mV) at different frequencies;  0.5 Hz (movie S2), 1 Hz (movie S3), 10 Hz (movie S4). The frame sequence (144, 65 and 46 frames, respectively) has been accelerated for illustrative purposes (original acquisition rate $1.00.s^{-1}$).

**movie S5. Neuronal activation assessed by GCaMP3 fluorescence in vivo.** In vivo response of L4 DRG neurons following mechanical (von Frey), cold (0°C) and heat (55°C) stimuli. Order of stimulation is von Frey (0–1 sec), cold (1–3 sec) and hot (3–5 sec). The frame sequence (120 frames) has been accelerated for illustrative purposes (original acquisition rate $1.54.s^{-1}$).

**movie S6. Neuronal activation following FCA treatment assessed by GCaMP3 fluorescence in vivo.** In vivo response of L4 DRG neurons to mechanical (pinch), cold (0ºC) and heat (55ºC) stimuli, 24 hours after intraplantar FCA administration. Order of stimulation is pinch (1–4 sec), cold (5–8 sec) and hot (10–13 sec). The frame sequence (170 frames) has been accelerated for illustrative purposes (original acquisition rate $1.54.s^{-1}$).

**movies S7 to S8. Neuronal activation following $PGE_2$ treatment assessed by GCaMP3 fluorescence in vivo.** In vivo response of L4 DRG neurons to heat (55ºC) stimuli before (movie S7) and 10 minutes after intraplantar $PGE_2$ administration (movie S8). The frame sequence (71 frames) has been accelerated for illustrative purposes (original acquisition rate $1.54.s^{-1}$).
